# Thermal Damping Applications of Coconut Oil–Silica Gels and Their Rheological Properties

**DOI:** 10.3390/gels11040261

**Published:** 2025-04-02

**Authors:** Jesús Javier Jiménez-Galea, Ana Isabel Gómez-Merino

**Affiliations:** 1 Department of Civil, Materials and Manufacturing Engineering, University of Malaga, Dr. Ortiz Ramos s/n, 29071 Malaga, Spain; j.jimenez@uma.es; 2 Department of Applied Physics II, University of Malaga, Dr. Ortiz Ramos s/n, 29071 Malaga, Spain

**Keywords:** coconut oil, fumed silica, thixotropy, viscosity, phase change material, gel, thermal damping, cooling

## Abstract

Phase change materials (PCMs) have the advantage of using the latent thermal heat as energy storage. Coconut oil (CO) has attracted much attention as PCM due to its high stability against oxidation. Although the viscosity of CO-based dispersions has extensively been studied, little research has been performed on time-dependent flow behaviors. In this work, the rheological properties of fumed silica dispersed in CO at fractions of 1, 2, 3, and 4 vol.% were investigated. All suspensions showed shear-thinning behavior, which became more Newtonian at temperatures above the phase change. The 3 and 4 vol.% suspensions showed gel-like microstructures. The thixotropic properties of the 3 vol.% suspension at 30 °C and 35 °C were mainly studied through stepwise changes in the shear rate. The results were consistent with thixotropic behavior, with a complete recovery of the microstructure. The sweep frequency of this sample demonstrated the dominance of the elastic modulus at both temperatures. Therefore, a thixoelastic nature of this gel could be inferred. This gel-like material flowed under high stress, providing superior thermal damping capabilities compared to conventional fluids. A reduction of 8.65 °C was confirmed after 30 min. of the laptop power supply operation.

## 1. Introduction

Conventional heat exchange fluids have generally lower thermal properties compared to solids, mainly, thermal conductivity. To address this limitation, nanofluids (NFs) have emerged as a transformative technology in industrial applications, such as manufacturing, engineering, electronics, and computing [1,2,3]. NFs are colloidal suspensions of nano-sized particles (<100 nm) in base fluids. NFs significantly enhance thermal properties and heat exchange efficiency in both conductive and convective heat transfer mechanisms [4]. The practical application of nanofluids is still under active investigation owing to the variability of results. Key factors governing their behavior and the heat transmission include the size, shape, and volumetric concentration of the nanoparticles, as well as the temperature, thermal conductivity, and flow dynamics of the base fluid [5,6]. The most important properties to consider in heat transfer applications are thermal conductivity and flow behavior, which are closely related to the stability of dispersions [6,7,8].

Suspensions based on phase change materials (PCMs) have the advantage of using the latent thermal heat as energy storage [9]. During the phase change transition, these PCMs exchange latent heat, melting and crystallizing, over several cycles. Typically, green phase change materials are found in vegetable oils (VOs). These substances are mainly composed of fatty acids extracted from oleaginous plants. They have a liquid appearance at room temperature, but their diverse chemical composition provides a wide range of phase change temperatures. In addition to being harmless, quite renewable, and biodegradable, the large recycling capability and easy disposal of VOs provide other advantages as PCMs. Their annual availability ensures environmental sustainability as opposed to PCMs derived from crude oil. Most vegetable oils are stored and when needed can be used in their virgin form. Although the production of some VOs in small quantities can be expensive, their final use does not require further thermal or chemical treatment, limiting their costs. In contrast, petrochemical PCMs usually are produced after several distillation and purification operations, increasing the final price. However, VOs are easily flammable and have low thermal conductivity, which are their main disadvantages [10,11,12]. Coconut oil (CO) has attracted much attention as PCM due to its high content of saturated fatty acids, which helps against oxidation. For that reason, the oxidative stability of CO is higher than other vegetable oils [13,14]. The addition of nanoparticles improves the thermal and flow properties of these base liquids. Nano additives, such as fumed silica, exhibit superior performance in thermal conductivity, thickening, thixotropy, and sag resistance with respect to the base fluids [15,16].

The rheological properties of fumed silica dispersed in vegetable oil play an important role in heat exchange applications. These colloidal suspensions can develop non-Newtonian behaviors, which should be taken into consideration [15,16]. The stability of the suspensions is of utmost importance because the thermal and flow properties could vary if the nanoparticles are not well dispersed. Several researchers have investigated the rheological properties of CO-based nanofluids [17,18,19,20]. They have mainly characterized the flow behavior in steady state. For example, viscosity curves as a function of shear rate have been fitted using well-known mathematical models. However, little research has been conducted on time-dependent flow behaviors. In some cases, suspensions can develop phenomena such as thixotropy or viscoelasticity. The study of these phenomena is of great interest in heat transfer applications. The use of refrigerants with thixotropic properties facilitates coolant flow by reducing its viscosity under shear conditions. If the microstructure is restored after standing, this fluid could be reused in multiple cycles. In processes such as painting, drilling, food processing, and pharmaceutical formulations, among others, thixotropy is critical for both processing and product performance [21,22,23,24].

In previous works [22], we have investigated the thermal properties of fumed silica in coconut oil. It was found that such suspensions form a strong microstructure at volume fractions higher than 3 vol.%, which could be defined as gels. In this work, the rheological properties of fumed silica suspensions in CO at volume fractions of 1, 2, 3, and 4 vol.% were explored. Steady state viscosity versus shear rate curves were performed over a wide range of temperatures for all the silica concentrations. The increase in viscosity due to the phase change was quantified with the power law mathematical model. The time-dependent rheological behavior, such as thixotropy and viscoelasticity, of the 3 vol.% gel was investigated via stepwise changes and the hysteresis loop. Finally, a thermal damping application of this gel was presented.

## 2. Results and Discussion

### 2.1. Characterization Studies

#### 2.1.1. Nanofluid Chemical Composition: Particle Size and Shape

**Figure 1:**
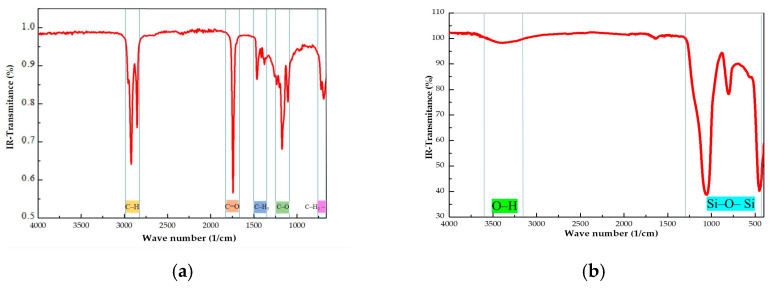
FTIR spectra of (**a**) virgin coconut oil (CO) and (**b**) fumed silica.

Most of the fatty acids that have been reported in virgin coconut oil are lauric acid (46.64–48.03%) and myristic acid (16.23–18.90%). In a smaller proportion, caprylic acid (7.19–8.81%) and capric acid (5.65–6.59%) are also present. Other substances such as polyunsaturated acids, tocopherol, and phenolic compounds are also found in very low quantities [25]. The FTIR of virgin coconut oil (CO) is depicted in Figure 1a. The absence of peaks near 3000 cm^−1^ and 1650 cm^−1^ indicate the lack of unsaturated fatty acids. The characteristic bands of the –C–O stretching vibration ester linkage at 1174 and 1105 cm^−1^ and the –C=O stretching at 1742 cm^−1^ are present. These are predictable bands of fatty acid ethyl esters. We also found the characteristic bands of alkyl chains, such as C–H (–CH_2_) stretching at 2924 cm^−1^ and 2851 cm^−1^; –C–H (–CH_2_, CH_3_) bending at 1465 cm^−1^; –C–H (–CH_3_) bending at 1378 cm^−1^; and –(CH_2_)_n_– at 720 cm^−1^. This IR spectrum agreed with other reported IR spectra of coconut oil [26,27]. Figure 1b shows the FTIR spectrum of fumed silica A200. Two peaks at 1061 cm^−^^1^ and 802 cm^−^^1^ could be distinguished, which are characteristics of the stretching vibration of the Si-O-Si structure. The shoulder found at 3300–3500 cm^−^^1^ can be assigned to the O–H stretching vibration of the silanol groups, Si-OH. A small peak at 1635 cm^−^^1^, due to the water molecules adsorbed onto the Si-O-Si bonds, was observed [28].

**Figure 2:**
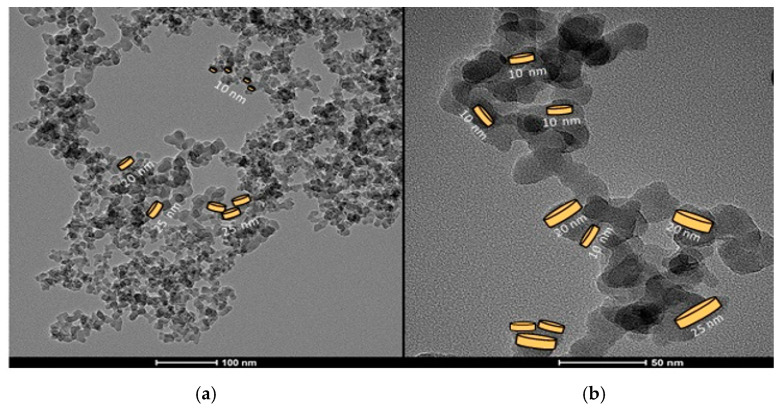
(**a**) TEM images of the silica powder A200 forming aggregates of fractal structures at two magnifications. (**b**) Platelet-like shapes of different sizes are highlighted in one of the fractal structures formed by the silica aggregates.

Figure 2 shows the TEM (transmission electron microscope) micrography of the silica powder A200 at two distinct magnifications—100 nm and 50 nm, respectively. The TEM image revealed an average diameter of 20 ± 5 nm. In Figure 2a, fractal structures of particles or groups of particles are shown, and some platelet-like shapes of different sizes are drawn. In Figure 2b groups of particles with fractal dimensions are presented.

#### 2.1.2. Differential Scanning Calorimetry (DSC)

**Figure 3:**
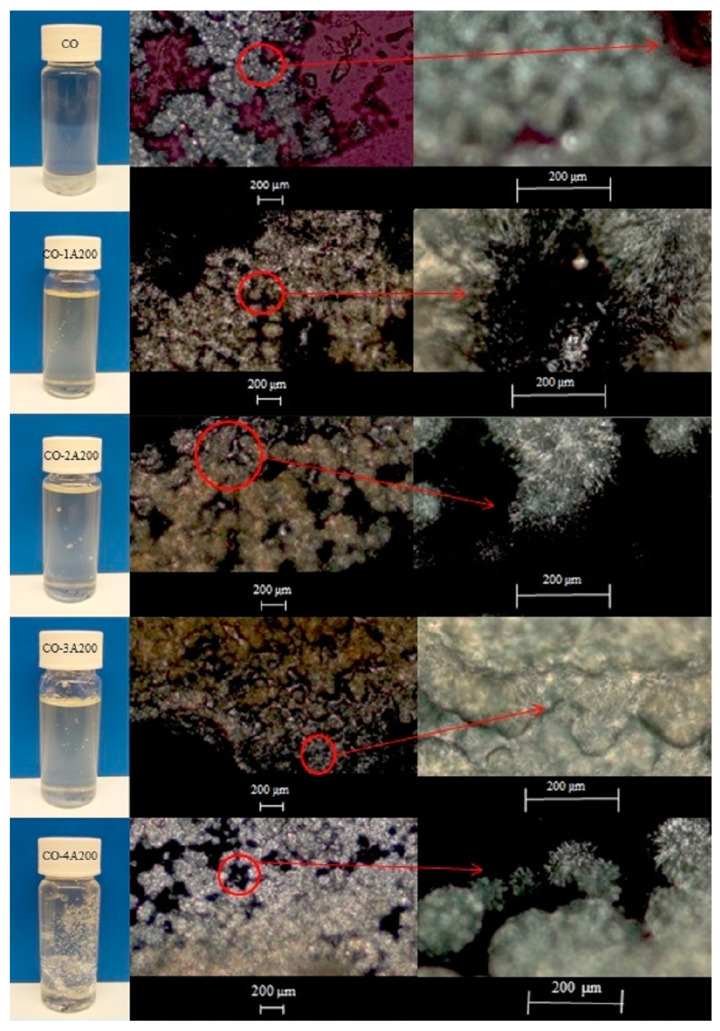
(**Left**) Suspensions of fumed silica A200 in coconut oil (CO) at 1, 2, 3, and 4 vol%. (**Right**) Polarized light optical microphotographs at two magnifications of the CO and the CO-A200 suspensions at 10 °C.

The evaluation of calorimetric properties is of paramount importance because additives may affect the phase change latent heat and the flow properties. Figure 3 (left) shows the base fluid CO (coconut oil) and the suspensions of silica A200 in CO at 1, 2, 3 and 4 vol.%. (Right), Polarized light optical microphotographs at two magnifications of the CO and the CO-A200 suspensions at 10 °C, when the coconut oil was crystalized. In this Figure, it can be demonstrated that the addition of silica particles strengthens the microstructure of the suspensions. The higher the particle loading the more solid-like structure was formed. The gel-like appearance is mainly observed at 3 and 4 vol% of fumed silica particles.

**Figure 4:**
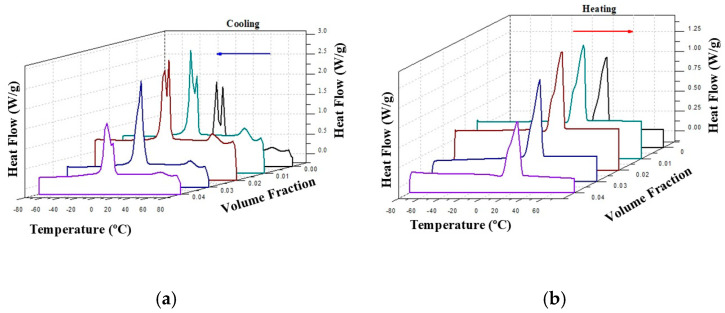
Two-dimensional DSC cooling (**a**) and heating (**b**) dynamic thermograms of CO base fluid and silica A200 in CO suspensions at various concentrations Same color means same samples.

Figure 4 presents the corresponding 3-D DSC cooling and heating dynamic thermograms of the CO–silica suspensions. Figure 4a shows the DSC cooling thermogram of coconut oil (CO) and the suspensions of silica in CO. The crystallization process of coconut oil is characterized by two peaks at −5.74 °C and 1.33 °C, respectively. The values for CO agree with others found in the literature [26]. These two peaks shift to higher temperatures and overlap with the addition of silica A200, becoming a shoulder at 3 and 4 vol% of silica, as demonstrated in Figure 4a.

However, the DSC heating thermogram of all the samples slightly changed with the addition of silica, as can be observed in Figure 4b. Contrary to the crystallization process in which heat conduction is the predominant heat exchange mechanism, in the melting process heat transfer involves mechanisms of both thermal conduction and natural convection. This complexity of the heat exchange process is reflected in the thermogram as a broad peak with the same phase change temperature for all the samples, around 24 °C. These thermograms were analyzed in previous work [22]. Table 1 shows the phase change latent heat of the four suspensions and the base liquid. A linear decrease in the phase change latent heat can be assumed. However, for heat exchange purposes the challenge should be an enhancement of c_p_ with the minimum reduction in latent heat exchange. The CO–3A200 (A200 silica dispersed in CO at 3 vol.%) suspension maintained a good latent heat value and good enhancement of the specific heat capacity with respect to the CO [22]. For clarity, the phase change temperatures are also displayed in Table 1. This table demonstrates how the crystallization temperature peaks shifted to slightly higher temperatures with the silica addition. Since colloidal suspensions are a mixture of solid and liquid, the solid phase could somewhat increase the crystallization temperature of the liquid phase.

### 2.2. Rheological Behavior

The flow properties of the CO–A200 suspensions provide very useful information for a better understanding of the microstructure of suspensions. This study was divided into steady and oscillatory shear tests.

#### 2.2.1. Viscosity Curves in Steady Shear

**Figure 5:**
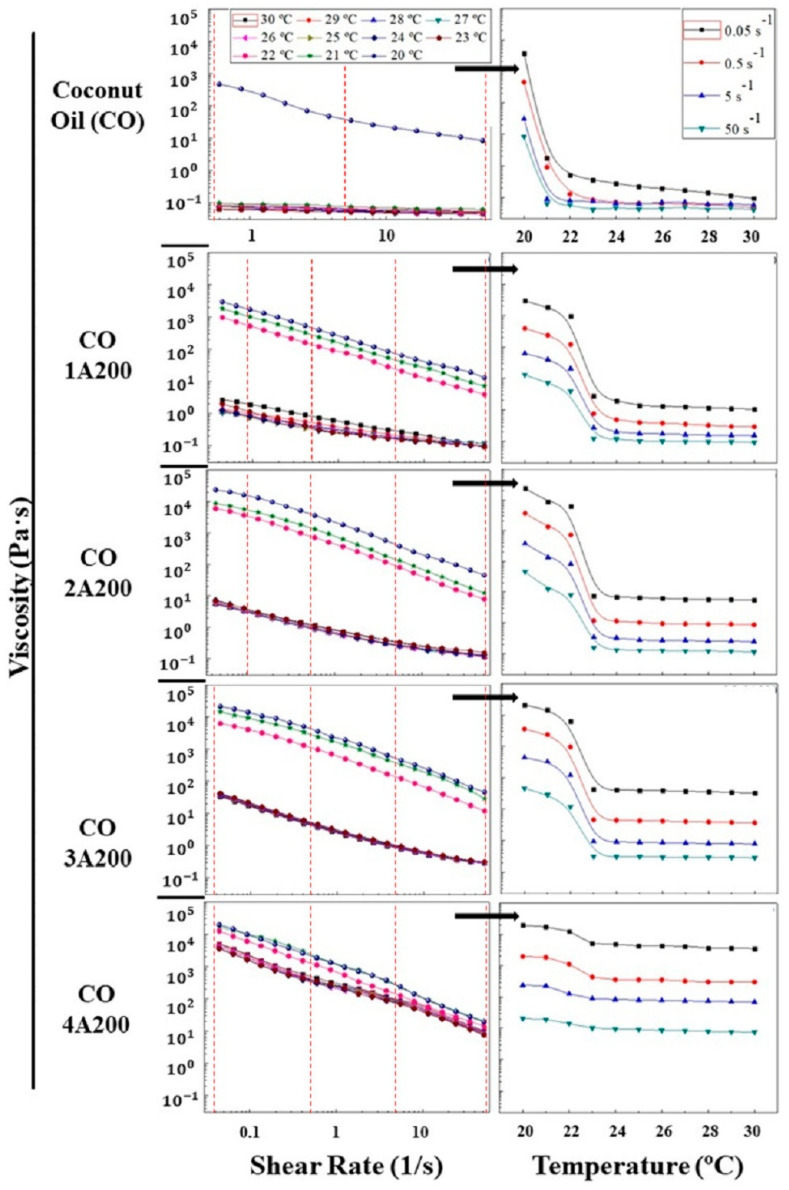
(**Left**) The viscosity versus shear rate curves in the temperature interval range of 20–30 °C. (**Right**) Viscosity versus temperature curves at different shear rates. This picture was obtained from the viscosity values at the shear rates indicated by the red dashed lines represented in the figure on the left.

The viscosity–shear rate curves in the interval of 20–23 °C are shown in Figure 5 (left). For all the temperatures, it can be observed that the viscosity decreases with the increase in shear rate. This behavior is known as shear-thinning flow. This phenomenon has been explained based on microstructure breakage by the action of the shear mechanical energy. In the case of colloidal suspensions, the microstructure disruption occurs with the alignment of particles. Although, the existence of a previous superstructure was not necessary to observe shear-thinning behavior. More recently, an entropic origin of the shear-thinning phenomena has been pointed out. That is, the decrease in the entropy change (entropic forces) because of small changes in the suspension microstructure [29,30]. The melting process (heating) caused a great reduction in the viscosity over 21 °C in coconut oil. But the effect of the phase change (melting or crystallization) on the suspension viscosities was diminished when increasing particle concentration. For example, in the suspension 4 vol.% the viscosity gap due to the phase change was less pronounced. While at a volume fraction of 3, 2, and 1 vol.% a big jump in viscosity during the phase change was observed, as shown in Figure 5 (left).

Figure 5 (right) represents the effect of temperature on the suspension viscosities at different shear rates. These curves were obtained from the viscosity–shear rate curves displayed in Figure 5 (left), as shown in this image. The red dashed lines of Figure 5 (left) represent the shear rates whose viscosities are represented in the figure on the right. Therefore, these viscosities are independent of the direction of the phase change, melting or crystallization, since all the viscosity curves on the left were recorded at a constant temperature. As explained above, the melting process reduced the viscosity of the samples, as can be seen in Figure 5 (right). That is, the increase in temperature produced a reduction in the viscosities of the suspensions. For all the shear rates analyzed, a decrease in viscosity was found during melting (heating), which was more noticeable in the base liquid and in the lower volume fractions (1, 2, and 3 vol.%). It can be observed that the 4 vol.% suspension experienced a smaller viscosity reduction during the phase change. In all the suspensions, the phase change temperature varied in the range of 20–23 °C. The decrease in viscosity by the action of the temperature can be explained by the growth of the Brownian motion (movement of particles through liquids); because of the increment of the kinetic energy of both solvent molecules and colloidal particles. The boost of the molecular motions reduces the friction between the liquid layers and the dissipated energy. In addition, particle motion can weaken the attractive forces (van der Waals) between particles. Both effects result in a reduction in the suspension viscosity [29]. After the melting process, temperature exerted a minor influence on all the suspension viscosities. This effect weakened with particle loading, as can be seen in Figure 5 (right). However, during the phase change, melting or crystallization, the flow behavior became non-Newtonian. At temperatures below the phase change the viscosity rose sharply, mainly at lower volume fractions (see 1, 2, and 3 vol.% curves, image below right).

Power law is a mathematical model (Equation (1)) [31] that better describes the viscosity curves exhibited in Figure 5 (left).

(1) $$\eta =K\times {\dot{\gamma }}^{n-1}$$

**Figure 6:**
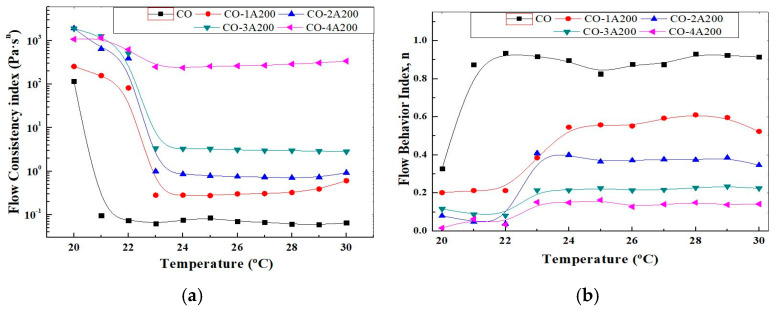
(**a**) Flow consistency index of CO–A200 suspensions versus temperature. Volume fraction effect. All uncertainties are below 5%. (**b**) Flow behavior index of CO–A200 suspensions versus temperature. Volume fraction effect. Both indexes are related according to Equation (1). All uncertainties are below 2%.

Equation (1) can be easily linearized representing the logη versus log$\dot{\gamma }$. The slope of this line is (n − 1), where n is the flow behavior index. The intercept is logK, where K is called the flow consistency index. It reflects the thickness or the fluid resistance to flow. K also provides a complete picture of the fluid response under several shear conditions, at a given temperature. The influence of particle concentration and temperature on K can be better visualized in Figure 6a. In this picture, the flow consistency index is displayed as a function of temperature for all the A200 concentrations. As it was explained before, K increases with volume particle concentration and reduces significantly over the phase change temperatures. This picture also confirms the small change in K with temperature at a 4 vol% of silica. From the rheological point of view, K is related to the microstructure of the suspensions. High K values could be associated with a more interconnected network, such as structured suspensions or gels. The CO–A200 nanofluids were proven to have a microstructure consisting of aggregates with fractal structures surrounded by layers of base fluid molecules. These particle clusters are formed when the attractive van der Waals forces between particles overcome the repulsive forces. The radius characterizing the aggregates becomes smaller with the addition of particles, which contributes to the strengthening of the nanofluid microstructure. Examples of structured suspensions or gels are 3 and 4 vol.% [22]. Figure 6b shows the effect of temperature on the flow behavior index, estimated from Equation (1). According to this picture, at temperatures below the phase change the suspensions are far from the Newtonian behavior. While at temperatures over the phase change the flow becomes more Newtonian at low volume fractions, as can be expected. At 3–4 vol% the change in the flow index with temperature is minor.

#### 2.2.2. Stepwise Changes Tests

**Figure 7:**
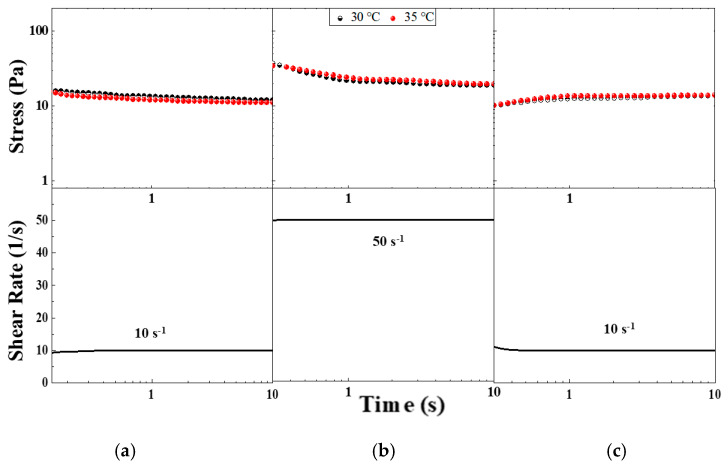
Stepwise changes in shear rate in CO–3A200 sample. (**a**) Stepwise increase test from rest to shear rate of 10 s^−1^, 30 °C and 35 °C curves. (**b**) Stepwise increase test from shear rate of 10 s^−1^ to shear rate of 50 s^−1^, 30 °C and 35 °C curves. (**c**) Stepwise decrease test from shear rate of 50 s^−1^ to 10 s^−1^, 30 °C and 35 °C curves.

Thixotropy could be defined as the rheological behavior characterized by a time-dependent decrease in viscosity caused by shear flow. This effect should be reversible when the flow is ceased. Therefore, the viscosity of these materials depends on the shear history. They could be shear-thinning and time-dependent, and they could also exhibit yield stress. Stepwise changes in shear rate provide a good basis for evaluating thixotropy behaviors. Figure 7 shows the stepwise changes in the shear rate of the CO–3A200 sample. The suspension is first sheared to remove any handling history. It is then left resting to recover the microstructure, which consists of a distribution of cluster sizes. The sample at rest is suddenly subjected to a constant shear rate of 10 s^−1^ for 10 s. The material response produces a stress overshoot which gradually decreases until reaching a steady state value. In Figure 7a, the corresponding curves at 30 °C and 35 °C of the step-down test are presented. If the shear rate increases to 50 s^−1^ (Figure 7b) in a step-up test the CO–3A200 suspension responds with a sudden rise in the stress followed by a gradual decay towards the steady value. If a rapid decrease in the shear rate to 10 s^−1^ is now applied a gradual stress growth response towards a constant value is observed (Figure 7c). The behavior shown in Figure 7 is consistent with a thixotropic response of the material. The effect of the shear flow was reversible with a complete recovery of the microstructure, as can be seen in Figure 7a,c [32,33]. The mechanism of thixotropy could be explained as follows. When the suspension is at rest a size distribution of flocs is formed. If mechanical energy is applied the sudden shear rate will normally decrease floc size because they are broken down by the hydrodynamic forces and the stress is reduced, as presented in Figure 7a at a shear rate of 10 s^−1^. If the shear rate increases at 50 s^−1^ (see Figure 7b) the mechanical energy will reduce the floc sizes and the suspension stress even more until a lower constant value is obtained. A stepwise decrease in the shear rate allows the dispersion to build up the microstructure again. The reduction in the mechanical shear energy can induce the cluster flocculation to recover the original microstructure. The mechanism by which the suspension recovers the former microstructure could be tentatively supported by the formation of hydrogen bonds between the particle silanol groups and the fatty acid esters of the coconut oil [34]. These bond formations could be facilitated by the sheared energy. Mechanical shear energy can not only cause floc breakdown but also induce flocculation to recover the original microstructure (see Figure 7c). Some examples of thixotropic material can be found in pharmaceutical products, such as topical hydrogel, ophthalmic and parenteral formulations [24], paints [21], and some food substances like gum, egg white, etc. [23].

#### 2.2.3. Hysteresis Loop

**Figure 8:**
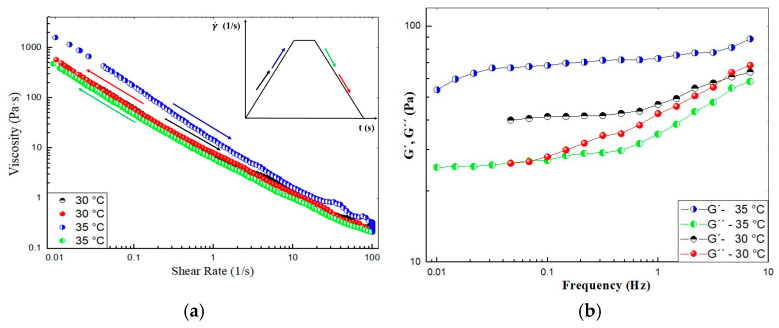
(**a**) Ramp of shear test of CO–3A200 gel at 30 °C and 35 °C. Each ramp lasts 37.5 s, respectively, and the plateau of shearing is performed at 100 1/s for 10 s. The arrow colors point out the direction of the ramp. (**b**) Frequency sweep test of CO–3A200 gel at 30 °C and 35 °C.

The hysteresis loop is a common phenomenon associated with thixotropy. It is a time-dependent test versus the viscosity curves recorded in steady state. It is observed in the viscosity curves upon ramping up the shear rate to a maximum value and then ramping it down to the initial value. Figure 8a shows the shear rate ramp of the CO–3A200 suspension at 30 °C and 35 °C. At 30 °C the hysteresis area is negligible. But at 35 °C, the viscosity is higher when increasing the shear rate than in the ramping down part of the cycle. This means that there is a hysteresis loop in the viscosity, which is consistent with thixotropic behavior [33]. This loop can be justified because the mechanical energy breaks down the suspension microstructure, reducing the viscosity when increasing the shear rate. In the second part of the cycle, when reducing the shear rate, the material has no time to build up structures to recover the initial viscosity. Figure 8b shows the frequency sweep in the linear viscoelastic region (LVR), γ = 0.005, from 0.01 to 5 Hz of CO–3A200 at 30 °C and 35 °C. At both temperatures, the G′, elastic modulus, is higher than the loss modulus, G″. This behavior is characteristic of gel-like materials [35]. Although it seems inconsistent that the G′ at 35 °C is higher than the G′ at 30 °C similar behavior is observed in Figure 8a. As was explained in Section 2.2.1, both indexes, K and n, were not greatly affected by temperature. Therefore, it could be assumed that the microstructure of this suspension was hardly influenced by thermal energy. This figure could reflect a certain elastic behavior at both temperatures. Therefore, this gel could be described as thixoelastic material [32,33].

### 2.3. Buffer Application

**Figure 9:**
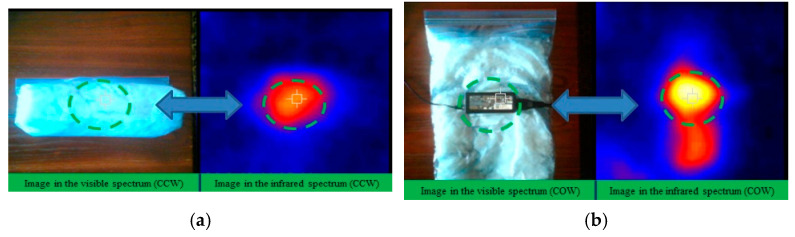
(**a**) (**Left**) The image of the visible spectrum of the Charger in the Close Wrapper. The circle in green dashed lines outstand the charger position which is shown on the right image. (**Right**) The image of the infrared spectrum of the Charger in the Close Wrapper (CCW). The circle in green dashed lines highlight the gradient of temperatures around the charger position. (**b**) (**Left**) The visible spectrum image of the Charger in the Open Wrapper (COW). The circle in green dashed lines outstand the position of the charger which is shown on the right image. (**Right**) Image of the infrared spectrum of the Charger in the Open Wrapper (COW). The circle in green dashed lines highlight the gradient of temperatures around the charger position.

The thermal properties of CO-A200 suspensions are shown in Figure 4 and discussed in Section 2.1.2. The flow properties of these suspensions are shown in Figure 5 and analyzed in Section 2.2. The combination of both analyses suggested that the CO–3A200 suspension was the most appropriate for heat exchange. To check the suitability of the CO–3A200 suspension as a heat exchanging material, a buffer test with a laptop charger was designed. A total of 300 mL of CO–3A200 was placed in a plastic bag (wrapping) and cooled in the refrigerator to a temperature around 15 °C. The computer charger was wrapped with the nanofluid bag, as is shown in Figure 9a, ensuring that the contact wrapper-charger was complete, to minimize heat losses and achieve effective heat transfer between both bodies. Thermographic pictures were taken to control the evolution of the temperature with time. In the first test, the thermal pictures were taken with the charger inside the wrapper, as shown in Figure 9a. The second test was performed with the wrapper open, as shown in Figure 9b. The third test was performed in the open air.

**Figure 10:**
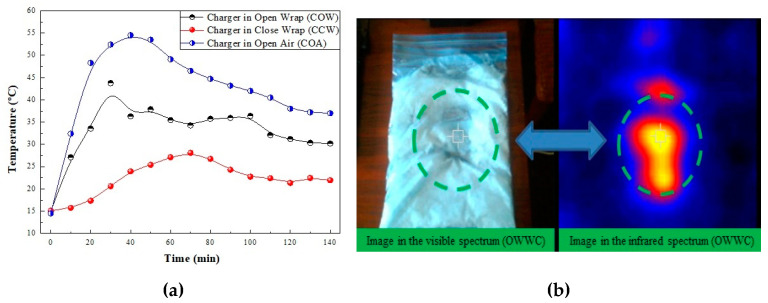
(**a**) The curves represent the evolution of temperature versus time when the charger is connected to the electricity supply over 140 min. The comparison of the charge process in open air (COA) with the charger covered by a close wrap (COW), as shown in Figure 9a, and the charger covered by an open wrap (CCW), as shown in Figure 9b. (**b**) Visible and infrared images of the wrapper after enclosing the charger for 140 min. (**Left**) The green dashed lines show how heat of the charger melted the coconut oil of the suspension which was inside the bag in contact with it. (**Right**) The same image in the infrared spectrum. The green dashed lines show the gradient of temperatures created around the charger.

Figure 10a shows the curves obtained in the three tests performed over 140 min, as described above. In the three tests, the thermal images were taken every 10 min. The test performed with the charger in open air (COA) showed a maximum temperature peak of 54.5 °C at 40 min, which began to slowly decrease to 37 °C at 140 min. The images taken of the outer side of the wrapper, named as charger in close wrapper (CCW), exhibited a temperature reduction of 9 °C (28 °C) at 70 min, which decreased to 22 °C at 140 min. The pictures taken of the charger on the open wrapper (COW) displayed a peak temperature of 38 °C at 50 min. This temperature reduced to 30 °C at 140 min. Figure 10b exhibits the visible and infrared images of the wrapper after enclosing the charger for 140 min. The green circle shows the shape of the charger when the heat of the power supply melted the coconut oil of the suspension, which was inside the bag in contact with it. When the heat transfer process was complete the nanofluid could be recycled to its initial state by cooling it in the refrigerator to the initial temperature of 15 °C. The heat exchange process can be repeated under the conditions initially described for more than 25 cycles. The stepwise tests demonstrated the recycling capacity of the CO–3A200 suspension. The thermal stability of this suspension was described in [22].

## 3. Conclusions

In this work, the rheological behavior of fumed silica in CO was discussed. The following conclusions can be drawn from the results obtained:

- The viscosity of the suspensions increased with the addition of fumed silica and reduced with temperature. The viscosities of all the suspensions experienced the greatest variation during the phase change in the base liquid, increasing during crystallization and decreasing during melting. However, above the phase change temperature the suspension viscosities demonstrated little variation with temperature.
- The 3 and 4 vol.% dispersions exhibited a gel-like microstructure. Stepwise changes in the shear rate of the 3 vol.% suspension at 30 °C and 35 °C revealed a trend which was consistent with a thixotropic response. However, the hysteresis loops showed a small area at 35 °C and a negligible area at 30 °C. These results could be interpreted as slight thixotropic behavior. For the two temperatures, the frequency sweep exhibited a predominance of the elastic modulus over the viscous modulus in the entire frequency range (0.01–8 Hz). Therefore, the CO–3A200 gel could be described as a thixoelastic material.
- In summary, the addition of 3 vol.% silica to coconut oil contributed to the formation of a strong and resilient internal structural suspension. This gel-like material flowed under high stress, facilitating optimal flow and rapid heat dissipation. The thixoelastic nature of this gel allowed for a reversible reconstitution of the microstructure under stress removal, thus ensuring the long-term use of the material. This rheological behavior conferred the CO–3A200 suspension a superior thermal damping capacity.

## 4. Materials and Methods

Coconut oil (CO) was purchased in Merck (KGaA, Darmstadt, Hesse, Germany). The density in the solid state was 0.916 ± 0.002 g/mL at 15 °C and in the liquid phase 0.9015 ± 0.0005 g/mL at 25 °C. Commercially available colloidal hydrophilic silica (A200) was purchased from Evonik Degussa Ibérica S.A. (Granollers, Barcelona, Spain). The real density was 2.318 ± 0.0012 g/cm^3^. The specific surface area BET (Brunauer–Emmett–Teller, City, State, Country) provided by the supplier, Evonik (Evonik industries, Hanau, Hesse, Germany) was 108 ≈ 200 ± 20 m^2^/g [22]. Nanofluids were prepared by adding silica powder to coconut oil at 25 °C in concentrations of 1, 2, 3, and 4 vol.%. The mixture was stirred in an Onilab OS40-Pro system (Labbox, Barcelona, Spain) at 750 rpm for 30 min. The samples were sonicated, with a frequency of 40 kHz and 50 W, at low vacuum for 30 min to disperse the powder and eliminate air bubbles (105 mb of maximum vacuum and 70 W).

A FTIR 6800FV model (Jasco, Madrid, Spain) was used to record the IR spectra in the range from 400 cm^−1^ to 4000 cm^−1^, with a standard resolution of 4 cm^−1^, as well as 64 accumulations per sample. Measurements were performed by attenuated total reflectance using the ATR ProOne accessory (Jasco, Madrid, Spain) and targeting in air, without the need to disperse or treat the samples. Electron micrographs (TEMs) were performed in a JEM-1400 TEM, JEOL Company, (Urbana, IL, USA), with a tungsten filament of 120 kV. The powder was dispersed in ethanol and some drops were left on Formvar (copper) grids. Thermal images were taken using a Testo model 872 camera (Testo SE & Co. KGaA, Neustadt, Baden-Wüttemberg, Germany), with a thermal sensitivity of 0.06 °C and resolution of 320 × 240 pixels, which provides infrared and/or real images in the range from −30 °C to 100 °C. Rheological tests were conducted in a Haake Mars 40/60 rheometer (ThermoFisher Scientific, Vreden, Germany) using a plate–plate geometry of 20 mm diameter made of stainless steel with a surface finish corresponding to a maximum roughness of Ra = 0.25 µm. A Peltier system controlled the temperature of the samples. Viscosity–shear rate curves were recorded in the interval of 0.01–100 s^−1^. The stepwise test was performed at shear rates of 10 s^−1^, 50 s^−1^, and –10 s^−1^, respectively. Each step lasted for 10 s. The frequency sweep was performed in the range of 0.01–10 Hz under a shear strain of 0.005. To eliminate the previous material history, a pre-shear stage was applied at 60 s^−1^ for 30 s followed by a 30 s rest time before the measurements of each test were recorded.

## Figures and Tables

**Table 1:** Latent heat values of A200 silica dispersed in CO. All the uncertainties are below 0.1%.

Parameters	0	0.01	0.02	0.03	0.04
ΔH_Melting_ (J/g)	102.4	98.8	94.5	89.2	84.3
T_peak_ (°C)	24.2	24.2	24.2	24.2	24.2
ΔH_Crystal_ (J/g)	97.2	94.1	90.1	86.2	82.9
T_peak_ (°C)	1.3; −5.7	3.7; −3.3	3.7; −1	3.7; −1	3.7; −3.3

## Data Availability

The original contributions presented in this study are included in the article, further inquiries can be directed to the corresponding author.
